# Sulfate ions promote Cnm-mediated aggregation in *Streptococcus mutans*: molecular dynamics insights into the oral–systemic connection

**DOI:** 10.3389/fmicb.2026.1967198

**Published:** 2026-10-23

**Authors:** Silvia Multari, Marco S. Nobile, Wenliang Zhu, Achille Giacometti, Paolo Cazzaniga, Giuseppe Pezzotti

**Affiliations:** 1 Department of Molecular Sciences and Nanosystems, Ca' Foscari University of Venice, Venice, Italy; 2 Department of Materials Chemistry, Kyoto Institute of Technology, Kyoto, Japan; 3 Biomedical Engineering Center, Kansai Medical University, Osaka, Japan; 4 Department of Environmental Sciences, Informatics and Statistics, Ca' Foscari University of Venice, Venice, Italy; 5 Department of Human and Social Sciences, University of Bergamo, Bergamo, Italy; 6 European Centre for Living Technology (ECLT), Ca' Bottacin, Venice, Italy

**Keywords:** bacterial aggregation, Cnm protein, molecular dynamics simulations, Raman spectroscopy, *Streptococcus mutans*

## Abstract

The collagen-binding protein Cnm from *Streptococcus mutans* has been implicated in cerebral microbleeds, a pathological condition frequently associated with ischemic stroke. Recent experimental evidence suggests that Cnm-mediated interactions may drive protein–protein aggregation, with the potential influence of ionic environment. In this work, we attempt to shed light on this issue by studying the molecular origin determinants of Cnm–Cnm association using a multiscale molecular dynamics approach. All-atom simulations were performed to evaluate the effect of sulfate ions (${\text{SO}}_{4}^{2-}$), selected based on the experimentally observed enrichment of sulfur-containing species in Cnm-positive *S. mutans*, on the stability of the complex. To extend the analysis to larger sizes and longer timescales, additional coarse-grained simulations using MARTINI force field were first implemented and validated against the atomistic reference, and subsequently used to explore the thermodynamics of protein–protein association. Our results indicate that sulfate ions significantly enhance the stability of the Cnm–Cnm complex, as evidenced by reduced fluctuations in the inter-protein distance, an increased number of intermolecular contacts, and a deeper minimum in the binding free-energy profile. These findings suggest that ionic screening can indirectly promote Cnm aggregation, providing physical mechanistic insights to aggregation processes potentially relevant to *S. mutans* pathogenicity.

## 1 Introduction

The oral cavity hosts a complex microbial ecosystem that interacts continuously with host tissues and the immune system, functioning both as a barrier and a gateway to systemic circulation (Sedghi et al., 2021; Pezzotti et al., 2026b). In recent years, the oral—systemic connection has gained increasing attention as evidence accumulated linking specific oral pathogens to extra-oral diseases (Peng et al., 2022). Among these, cerebrovascular disorders such as ischemic stroke have emerged as conditions potentially influenced by oral microbial factors, prompting a shift toward more integrative models of pathogenesis (Fu et al., 2025; Tonomura et al., 2016).

Within the oral microbiota, *Streptococcus mutans* is classically recognized for its role in dental caries (Loesche, 1986). However, a distinct subset of strains expressing the collagen-binding protein Cnm (referred to as Cnm-positive *S. mutans*, henceforth) has been implicated in systemic vascular pathology (Abranches et al., 2009; Nakano et al., 2011; Kesavalu et al., 2012). This virulence factor enables bacterial adherence to collagen-rich tissues, including exposed components of the vascular endothelium, thereby facilitating interactions beyond the oral cavity (Mieher et al., 2025; Xiao and Li, 2025). The presence of Cnm-positive strains in the bloodstream has been associated with endothelial dysfunction and increased vascular vulnerability (Leonov et al., 2024; Avilés-Reyes et al., 2017). Emerging evidence suggests an association between Cnm-positive *S. mutans* and cerebrovascular/cardiovascular disorders (Nakano et al., 2011; Nomura et al., 2020; Pezzotti et al., 2026a,b,c). Following transient bacteremia, Cnm-mediated adhesion to collagen-rich tissues may facilitate bacterial interactions with damaged or inflamed vascular surfaces (Miller et al., 2015). However, a full understanding of the molecular mechanisms linking Cnm-associated phenotypes to systemic vascular effects is still lacking.

Beyond cerebrovascular complications, *S. mutans* has also been associated with other systemic conditions, including infective endocarditis and atherosclerosis, further highlighting the potential relevance of oral pathogens in extra-oral disease. Cnm-positive *S. mutans* may also modulate systemic inflammatory responses (Shiga et al., 2021). The activation of immune pathways, along with the release of pro-inflammatory cytokines, can contribute to a pro-thrombotic state and endothelial dysfunction, which are both recognized risk factors for ischemic stroke (Theofilis et al., 2021). Clinical and epidemiological studies have reported higher prevalence rates of Cnm-positive strains in patients with a history of stroke, supporting a potential association, although causality remains to be fully established (Nomura et al., 2020).

Despite growing interest in the systemic implications of Cnm-positive *S. mutans*, the molecular determinants underlying its enhanced aggregation phenotype remain incompletely understood. In this study, we focus specifically on Cnm–Cnm association and investigate how the ionic environment may modulate the stability of the protein–protein complex. By integrating molecular dynamics simulations with previously reported microscopy and Raman spectroscopic observations, we examine Cnm self-association as one molecular mechanism potentially contributing to the aggregation phenotype of Cnm-positive *S. mutans*. While this study does not directly model bacterial adhesion to vascular tissues or stroke pathogenesis, it still provides a molecular-level characterization of Cnm–Cnm interactions that may contribute to understanding the broader biological phenotype.

## 2 Materials and methods

### 2.1 Molecular dynamics simulations

#### 2.1.1 System preparation

To understand the effect of the presence of sulfate ions on the stability of the system, in this study the Cnm–Cnm complex will be studied in aqueous solution with and without the addition of sulfate ions (Cnm–Cnm + ${\text{SO}}_{4}^{2-}$). The crystal structure of the Cnm dimer was obtained from the RCSB Protein Data Bank (PDB ID: pdb_00007lgr) (Mieher et al., 2025). The protein complexes were placed in a cubic simulation box of side length 10 nm and solvated with explicit SPC/E, for the atomistic simulations (Berendsen et al., 1995), and standard MARTINI water beads for the coarse-grained ones (Souza et al., 2021). For the sulfate-containing system, ${\text{SO}}_{4}^{2-}$ ions were added to reach a concentration of 1 μM. This concentration should be interpreted as a condition designed for simulations to probe the molecular effect of sulfate on Cnm–Cnm association rather than as a direct reproduction of the *in vivo* ionic environment. In both systems, Na^+^ and Cl^−^ ions were added to neutralize the total charge and to reproduce a physiological salt concentration of 0.15 M. These structures were used as the starting configurations for both the all-atom and coarse-grained simulations described below.

Snapshots of the initial configurations of both all-atom (left panel) and coarse-grained (right panel) calculations are shown in Figure 1.

**Figure 1:**
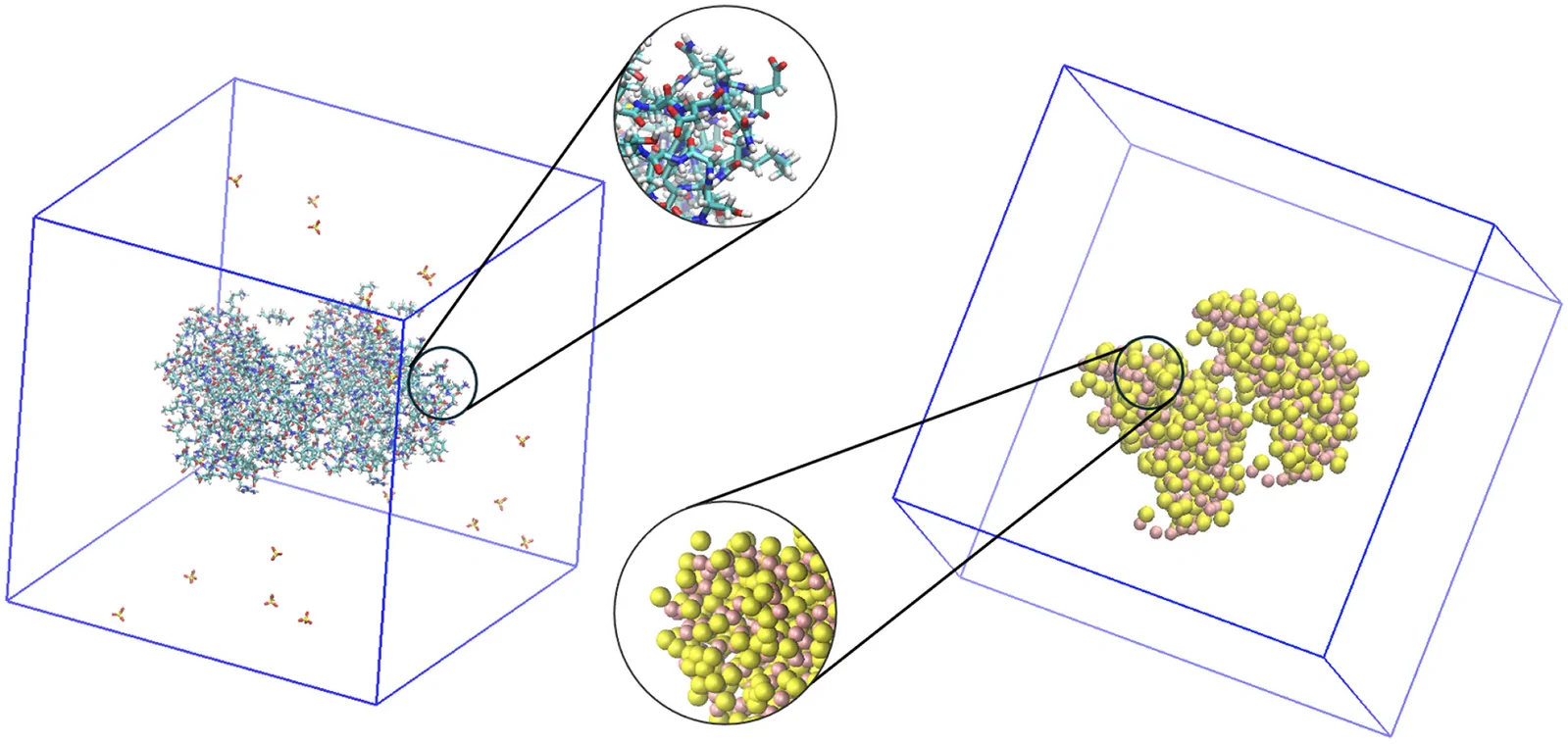
Comparison of the starting configurations between the All-Atom and Coarse-Grained models.

#### 2.1.2 All-atom molecular dynamics simulations

All-atom molecular dynamics (MD) simulations were performed using GROMACS (version 2022.3) (Plimpton, 1995). The Amber03 force field was used to model protein interactions (Duan et al., 2003). In this force field, the total potential energy of the system is expressed as a sum of bonded and nonbonded contributions:


$$\begin{array}{l} U=\underset{\text{bonds}}{\sum }\frac{1}{2}k_{r}{\left(l_{i}-l_{i,0}\right)}^{2}+\underset{\text{angles}}{\sum }\frac{1}{2}k_{\text{Θ}}{\left({\text{Θ}}_{i}-{\text{Θ}}_{i,0}\right)}^{2}\\ \;+\underset{\text{dihedrals}}{\sum }\frac{1}{2}k_{\psi }\left[1+\cos\left(n\psi +\text{Υ}\right)\right]+\underset{\text{impropers}}{\sum }\frac{1}{2}k_{\omega }{\left(\omega -\omega_{0}\right)}^{2}\\ \;+\underset{i<j}{\sum }4\epsilon_{ij}\left[{\left(\frac{\sigma_{ij}}{r_{ij}}\right)}^{12}-{\left(\frac{\sigma_{ij}}{r_{ij}}\right)}^{6}\right]+\underset{i<j}{\sum }\frac{q_{i}q_{j}}{r_{ij}}. \end{array}$$ (1)

In Equation 1, the first four components are bonded potentials that describe bonds, angles, proper dihedrals, and improper torsions of the covalent structure, respectively. The last two terms run over all pairwise atoms *i* and *j* separated from each other by a distance *r*_*ij*_ = |**r**_*j*_−**r**_*i*_| and describe the non-bonded interactions. The bond stretching and bond bending (1^st^ and 2^nd^ terms in Equation 1) model the energetic change accompanying the deformations of the bond lengths *l* and bond angles Θ from their respective equilibrium values *l*_0_ and Θ_0_. These two interactions are mainly computed using a harmonic-like potential with force constants *k*_*r*_ and *k*_Θ_. The third and fourth terms in Equation 1 account for bond rotations. Here *k*_ψ_ is the height of the rotational barrier associated with the proper dihedral angle ψ characterized by the torsional angle phase Υ for each Fourier component *n* (periodicity). Improper torsions ensure planarity in aromatic rings and allow distinguishing molecules from their mirror images, in which *k*_ω_ is the force constant for the improper dihedral ω going up and down its equilibrium position ω_0_. The last two components of Equation 1 describe the van der Waals repulsive (at short distance, *r*^-12^ term) and attractive (at long distance, *r*^-6^ term) pairwise atomic forces between *i* and *j* shown as Lennard-Jones 12-6 potential, and the electrostatic interactions. The variables *q*_*i*_ and *q*_*j*_ are the partial charges on pairwise atoms *i* and *j* separated by the distance *r*_*ij*_; σ_*ij*_ is the distance at which the Lennard-Jones potential is zero, and ϵ_*ij*_ is the well depth.

Standard Amber03 parameters provided in GROMACS (amber03.ff) were used for the protein and solvent. For sulfate ions, the oxygen atoms were described using existing Amber03 parameters, while a dedicated sulfur atom type (SO) was introduced using previously reported parameters, with a partial charge of +2*e* and Lennard–Jones parameters σ = 0.355 nm and ϵ = 1.0465 kJ mol^−1^ (Mamatkulov et al., 2018).

Energy minimization was carried out using the steepest descent algorithm with a maximum of 50,000 steps. Periodic boundary conditions were applied in all three spatial directions (pbc = xyz). Neighbor searching employed the Verlet cutoff scheme, with short-range electrostatic and van der Waals cutoffs of 1.0 nm. Long-range electrostatic interactions were treated using the Particle Mesh Ewald (PME) method with cubic interpolation of order 4 and a Fourier grid spacing of 0.16 nm. Van der Waals interactions were corrected for both energy and pressure.

Equilibration and production simulations used the leap-frog integrator (md) with a time step of 2 fs. All bonds involving hydrogen atoms were constrained using LINCS. Temperature was controlled using the velocity-rescale thermostat (modified Berendsen) applied separately to protein and non-protein groups, with a reference temperature of 300 K and a time constant of 0.1 ps for both groups. Pressure was controlled with the Parrinello–Rahman barostat in isotropic mode at 1 bar, with a time constant of 2 ps and a water compressibility of 4.5 × 10^−5^ bar^−1^. Coordinates were saved every 10 ps.

Equilibration was performed first in the NVT ensemble for 100 ps, followed by 100 ps in the NPT ensemble, using the parameters described above. Production simulations were carried out for 100 ns starting from the equilibrated structures, with velocities not regenerated to ensure continuity from the equilibration stage, and with all constraints removed to enable proper sampling. The 100 ns production length was selected to provide an atomistically resolved reference trajectory for assessing the structural stability and intermolecular interactions of the Cnm–Cnm complex, while maintaining a computationally tractable system size. This timescale was considered sufficient for the purpose of comparing the sulfate-containing and sulfate-free systems at the atomistic level, rather than for exhaustively sampling protein–protein association and dissociation events.

#### 2.1.3 MARTINI coarse-grained molecular dynamics simulations

Coarse-grained (CG) molecular dynamics simulations were performed using the MARTINI 3 force field (version martini3001) (Souza et al., 2021) in combination with GROMACS (version 2022.3). The atomistic structure of the protein complex was converted into its coarse-grained representation using martinize2 (Kroon et al., 2025). Secondary structure assignment was obtained using DSSP (Hekkelman et al., 2025), and an elastic network was applied to the protein backbone. Hydrogen atoms were ignored during the mapping procedure. During equilibration, position restraints were applied to the protein with a force constant of 1,000 kJ mol^−1^ nm^−2^.

Energy minimization was carried out using the steepest descent algorithm for 5000 steps until reaching a convergence tolerance of 10^−4^ kJ mol^−1^ nm^−1^ (Figure 2). Neighbor searching was performed using the Verlet scheme with a cutoff distance of 1.4 nm and a Verlet buffer tolerance of 0.005. Electrostatic interactions were treated using the reaction-field-zero method with a Coulomb cutoff of 1.2 nm and a relative dielectric constant ϵ_*r*_ = 15. Van der Waals interactions were modeled using a cutoff scheme with a potential-shift modifier, with a cutoff at 1.2 nm and a switching function starting at 0.9 nm. Periodic boundary conditions were applied in all directions. No bond constraints were applied. Temperature coupling during minimization was performed using the Berendsen thermostat at 300 K with a coupling constant τ_*t*_ = 1.0 ps.

**Figure 2:**
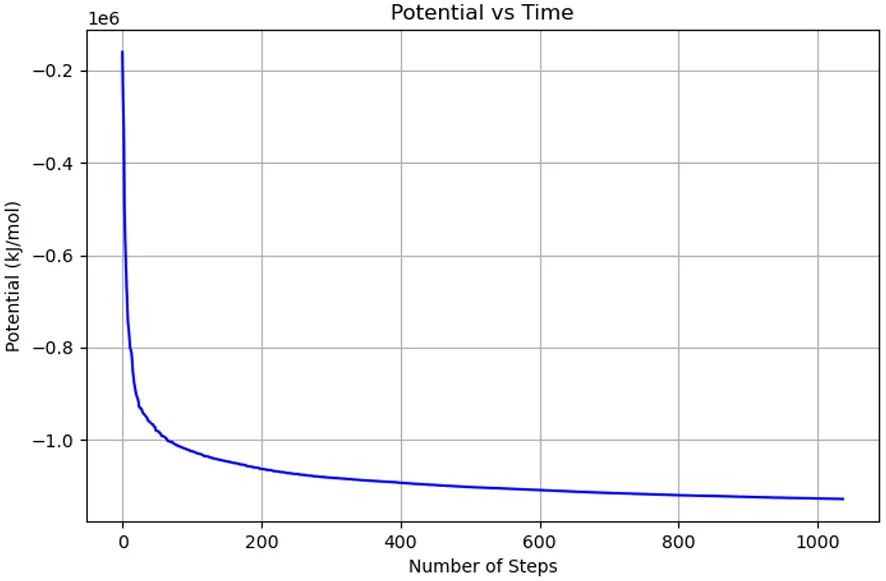
Potential energy profile during energy minimization.

Equilibration was performed in the NPT ensemble for 10 ns using a time step of 20 fs. Temperature was maintained at 300 K using the velocity-rescale (v-rescale) thermostat with a coupling constant τ_*t*_ = 1.0 ps, applied separately to protein and non-protein groups. Pressure was controlled at 1 bar using the C-rescale barostat under isotropic coupling conditions, with a coupling constant τ_*p*_ = 12 ps and a compressibility of 1 × 10^−4^ bar^−1^. Electrostatic interactions were treated using the reaction-field-zero scheme with a cutoff of 1.2 nm and ϵ_*r*_ = 15, while van der Waals interactions were described using a cutoff at 1.2 nm with potential shifting. Neighbor searching employed the Verlet scheme with *r*_list_ = 1.4 nm. Initial velocities were generated from a Maxwell–Boltzmann distribution at 300 K, and reference coordinate scaling was applied to the center of mass of restrained groups.

The CG production time was extended to 1 μs to exploit the reduced computational cost of the MARTINI representation and to sample the slower relative motions of the two proteins over a timescale one order of magnitude longer than the atomistic simulations. This extended trajectory was used to characterize the persistence of the protein–protein association and to sample a broader range of inter-protein separations and interface configurations required for the structural and thermodynamic analyses described below.

#### 2.1.4 Trajectory analysis

Trajectory analyses were performed using tools included in the GROMACS package (version 2022.3) as well as custom Python scripts. Prior to analysis, periodic boundary conditions were removed, and trajectories were re-centered on the protein complex.

Structural stability of the systems was evaluated by calculating the root-mean-square deviation (RMSD) of backbone atoms with respect to the initial structure. Inter-protein separation was monitored by measuring the center-of-mass (COM) distance between the two Cnm monomers over time. Analyses were performed over the 100 ns of the all-atom simulations and over the full 1 μs of the coarse-grained simulations to ensure adequate sampling.

Protein–protein interactions were further characterized by computing intermolecular contacts, defined as pairs of atoms belonging to different proteins within a cutoff distance of 0.5 nm. For coarse-grained simulations, contact frequencies were normalized to account for the reduced degrees of freedom relative to the all-atom representation, allowing direct comparison of interface interactions.

The Potential of Mean Force (PMF) along the inter-protein center-of-mass (COM) distance *r* was calculated from the probability distribution of sampled distances according to


$$\begin{array}{l} \text{PMF}\left(r\right)=-k_{B}T\ln\;g\left(r\right), \end{array}$$ (2)

where *g*(*r*) is the radial distribution function of COM distances obtained from the trajectory and *k*_*B*_*T* is the thermal energy at 300 K. Under the assumption that the molecular dynamics trajectory provides equilibrium sampling of the configurational space, the PMF obtained from this relation represents the reversible free-energy profile along the chosen reaction coordinate. A similar strategy has been successfully exploited in similar contexts (Foumthuim et al., 2024).

To assess the dependence of these profiles on sampling time, the analysis was repeated over cumulative trajectory windows of 0–250, 0–500, 0–750, and 0–1000 ns. Because the unbiased trajectories do not provide exhaustive equilibrium sampling of repeated association and dissociation events, these profiles are interpreted as qualitative descriptors of the sampled interaction landscape rather than as absolute thermodynamic free-energy profiles.

The histogram required for computing *g*(*r*) was computed over the full production trajectory using uniformly spaced bins along the COM distance coordinate. The resulting PMF profile was shifted such that its minimum corresponds to zero free energy, facilitating comparison between different systems.

The dissociation free energy Δ*G* was estimated from the PMF profile as the free-energy difference between the bound state and the unbound plateau. The bound state corresponds to the global minimum of the PMF at short COM distances, representing the stable protein–protein complex. The unbound reference state was defined as the region at large separations where the PMF becomes approximately constant, indicating negligible interactions between the two proteins. In practice, the free energy of the unbound state was obtained by averaging the PMF values over the largest sampled separations (top 10% of the COM distance range). The dissociation free energy was then calculated as


$$\begin{array}{l} \text{Δ}G=G_{\text{unbound}}-G_{\text{bound}}, \end{array}$$ (3)

where *G*_bound_ corresponds to the PMF minimum and *G*_unbound_ is the average PMF value in the plateau region at large distances.

The solvent-accessible surface area (SASA) was calculated using the gmx sasa tool in GROMACS with a probe radius of 0.14 nm, corresponding to a water molecule. SASA was computed for the full protein complex as well as for the individual monomers after separating them in space. This is known to be a very good indicator of the on-going self-assembly process (Cezar et al., 2026).

The buried surface area (BSA) upon complex formation was estimated as


$$\begin{array}{l} \text{ΔSASA}=\text{SAS}{\text{A}}_{\text{monomers}}-\text{SAS}{\text{A}}_{\text{complex}}, \end{array}$$ (4)

where SASA_monomers_ is the sum of the solvent-accessible surface areas of the two proteins in the unbound state and SASA_complex_ is the SASA of the bound complex.

All trajectory visualizations were generated using VMD (Humphrey et al., 1996).

## 3 Results

### 3.1 Microscopy observations and Raman spectroscopic evidences

Laser micrographs of Cnm-negative and Cnm-positive strains of *Streptococcus mutans* grown in the absence of sucrose, a condition that prevents biofilm formation, are shown in Figure 3 from a previous work (Pezzotti, 2026). Under these environmental circumstances, a clear morphological difference emerges: cells of the Cnm-positive strain (a) display a stronger tendency to cluster, forming larger and more numerous aggregates compared to the more dispersed Cnm-negative cells (b). This observation suggests an enhanced adhesive capability in the Cnm-positive strain, likely reflecting differences in cell surface composition at the molecular level. Such findings provide initial structural evidence distinguishing the two strains, which is further supported by the spectroscopic and computational analyses discussed in the following section.

**Figure 3:**
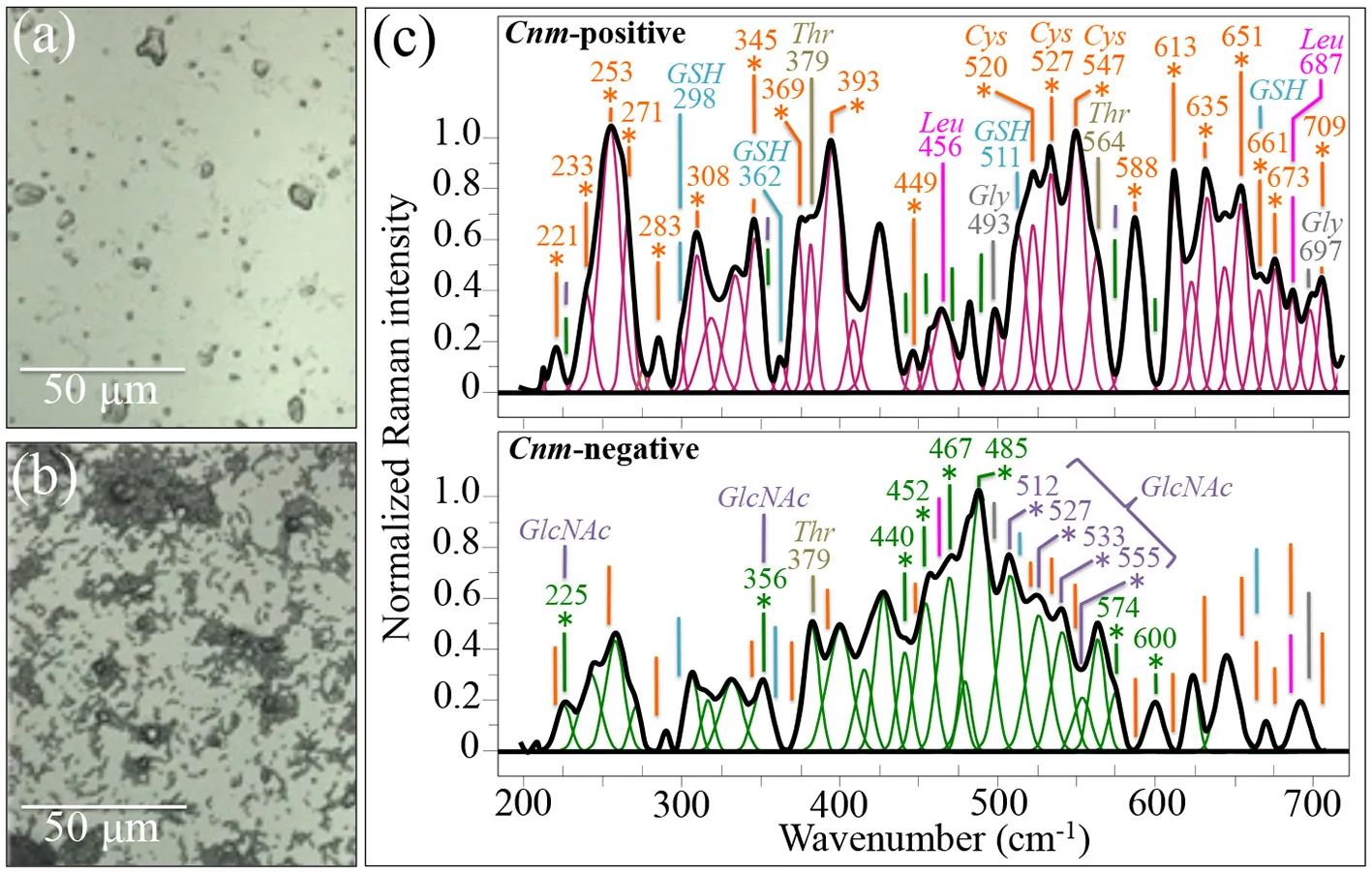
**(a, b)** Laser micrographs of Cnm-negative **(a)** and Cnm-positive **(b)** strains of *Streptococcus mutans* grown in the absence of sucrose, highlighting the stronger aggregation tendency of Cnm-positive cells compared to the more dispersed Cnm-negative counterpart. **(c)** Averaged Raman spectra in the 200–700 cm^−1^ region for Cnm-positive (upper trace) and Cnm-negative (lower trace) bacteria, showing pronounced molecular differences, including enhanced S–S stretching signals in the Cnm-positive strain (Pezzotti, 2026).

Figure 3c shows the averaged Raman spectra collected in the 200–700 cm^−1^ low-wavenumber region for Cnm-positive (upper spectrum) and Cnm-negative (lower spectrum) bacteria (Pezzotti et al., 2026a). This spectral window reveals substantial molecular differences between the two strains. Specifically, 25 Raman bands (out of 39 total) are clearly detectable in the Cnm-positive spectrum but are absent or significantly reduced in the Cnm-negative counterpart. Such marked disparity highlights profound differences in biochemical composition. Notably, the presence of the Cnm protein enhances the signal near 527 cm^−1^, which corresponds to S–S stretching vibrations associated with disulfide bonds in cysteine (Cys) residues. Additional contributions from disulfide vibrations are observed near 511 and 547 cm^−1^, although the former partially overlaps with signals from glutathione (GSH).

Among the spectral features unique to the Cnm-positive strain, a large subset (15 bands) can be attributed to oxidized sulfur species (Pezzotti et al., 2026a). These signals likely arise from a mixture of metabolic intermediates and degradation products related to sulfur metabolism. A particularly intense band at 253 cm^−1^ is consistent with S–S stretching in disulfide-containing ions. Given that both strains were analyzed under identical experimental conditions, these differences point to altered sulfur metabolism in the Cnm-positive strain, possibly involving reduced activity of methionine sulfoxide reductases. Such a shift may favor the accumulation of oxidized sulfur compounds at the cell surface, thereby enhancing adhesion. In this context, sulfur-containing molecules may contribute directly to surface “stickiness,” reinforcing the adhesive phenotype associated with the Cnm protein. Additional bands at 635 and 651 cm^−1^ are consistent with contributions from indoxyl sulfate, further supporting the enrichment of sulfur-related species.

Other Raman bands observed exclusively in the Cnm-positive strain can be linked to amino acids. Peaks near 456 and 687 cm^−1^ are attributable to leucine (Leu), although the latter may overlap with indoxyl sulfate contributions. Signals at approximately 493 and 697 cm^−1^ are mainly associated with glycine (Gly), while a band at 564 cm^−1^ corresponds to threonine (Thr). Additional weaker features at 298 and 362 cm^−1^ are tentatively assigned to vibrational modes of GSH, although its stronger characteristic bands are masked by overlapping sulfur-related signals.

In contrast, the Cnm-negative strain exhibits eight distinct Raman bands (225, 356, 440, 452, 467, 485, 574, and 600 cm^−1^) that are not present in the Cnm-positive spectrum. These features are primarily associated with mono- and polysaccharides. In particular, several bands within the 350–600 cm^−1^ region (356, 440, 485, 574, and 600 cm^−1^) are characteristic of glycogen, with the 485 cm^−1^ peak serving as a well-recognized marker due to minimal spectral overlap. Additional contributions in this region are consistent with D-glucosamine and N-acetyl-D-glucosamine (GlcNAc), key components of the bacterial peptidoglycan matrix. Signals at 225 and 467 cm^−1^ also reflect the presence of glucose- and sucrose-related structures.

Taken together, these spectroscopic features indicate that the Cnm-negative strain has a greater capacity for glycogen accumulation and carbohydrate storage, whereas the Cnm-positive strain appears to prioritize the expression of virulence-associated factors, including the Cnm protein and sulfur-rich surface components (Pezzotti et al., 2026a; Pezzotti, 2026). This metabolic divergence suggests distinct adaptive strategies: the Cnm-positive strain may enhance its invasive potential through increased adhesion, while the Cnm-negative strain invests more heavily in energy storage. Consistent with this interpretation, the enrichment of surface-associated Cnm protein and sulfur compounds in the Cnm-positive strain may reduce the reliance on a robust peptidoglycan layer, potentially leading to a simplified cell wall structure.

### 3.2 Molecular dynamics simulations

The system described above is clearly too complex to be tackled by computer simulations in its fully-fledged conformation. However, it is possible to single out some specific components of the system for which *in silico* analysis may provide additional insights on the experimental findings. In particular, it is possible to understand the effect of the presence of the sulfate ions on the complex stability, as discussed below.

#### 3.2.1 Validation of the coarse-grained model

To extend the study of the protein complex to the microsecond timescale, we first validated the Coarse-Grained (CG) MARTINI model against a 100 ns All-Atom (AA) reference simulation, using the complex in the presence of sulfate ions. This is a common strategy and has been successfully used before in similar contexts (Carrer et al., 2020). Comparison of the radial distribution function *g*(*r*) between the centers of mass (COM) of the two proteins shows good agreement between the two models, with both converging to an equilibrium inter-protein distance between 2.2 and 2.6 nm (Figure 4A). Analysis of the relative intermolecular contacts, normalized to account for the reduced degrees of freedom in the CG representation, further confirms that the interface interactions are preserved and that no structural drift or non-specific sliding occurs over the validation window (Figure 4B).

**Figure 4:**
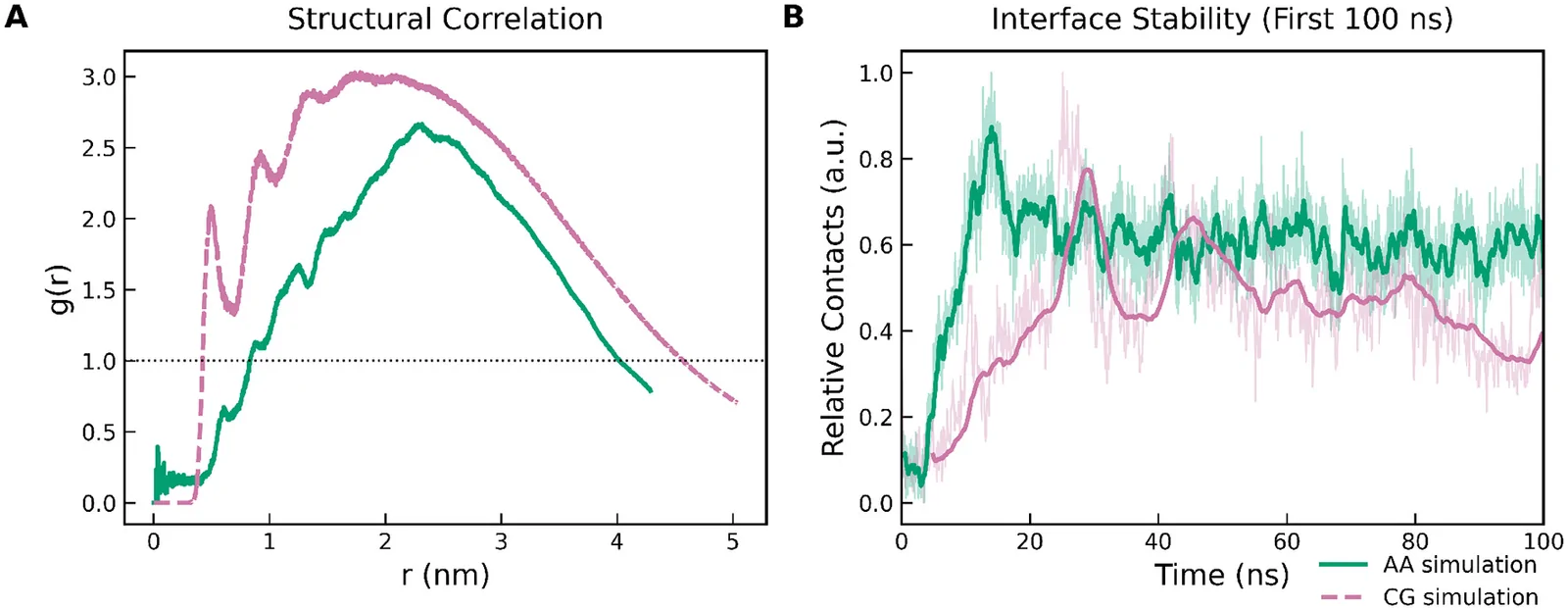
Validation of the Coarse-Grained (CG) model against the All-Atom (AA) reference simulation. **(A)** The radial distribution function *g*(*r*) demonstrates structural consistency. **(B)** The normalized intermolecular contacts show stable interface dynamics over the simulation.

These results confirm the validity of the CG model as a reliable representation of the system.

#### 3.2.2 Effect of sulfate ions on complex stability

Having established this structural and dynamical consistency, we can therefore rely on the CG model to analyze longer simulations and extract thermodynamic properties, taking advantage of its significantly lower computational cost in pushing the calculations to longer time scales and larger systems. Henceforth, all further analyses of binding stability, interface dynamics, and energetic profiles will therefore be based on the CG simulations, providing a reliable and computationally efficient framework to assess the stabilizing role of sulfate ions.

We then performed extended simulations and extracted thermodynamic and structural properties using CG simulations, and subsequent analyses of binding stability, interface dynamics, and energetic profiles are based on these CG simulations. The structural stability of the complex was first assessed through the time evolution of the center-of-mass (COM) distance (Figure 5A). In the presence of sulfate ions (${\text{SO}}_{4}^{2-}$), the proteins remain in close proximity, with a mean COM distance of 29.35 Åand a minimum of 18.35 Å. In contrast, the sulfate-free system displays a significantly larger mean distance (76.70 Å), indicative of partial dissociation.

**Figure 5:**
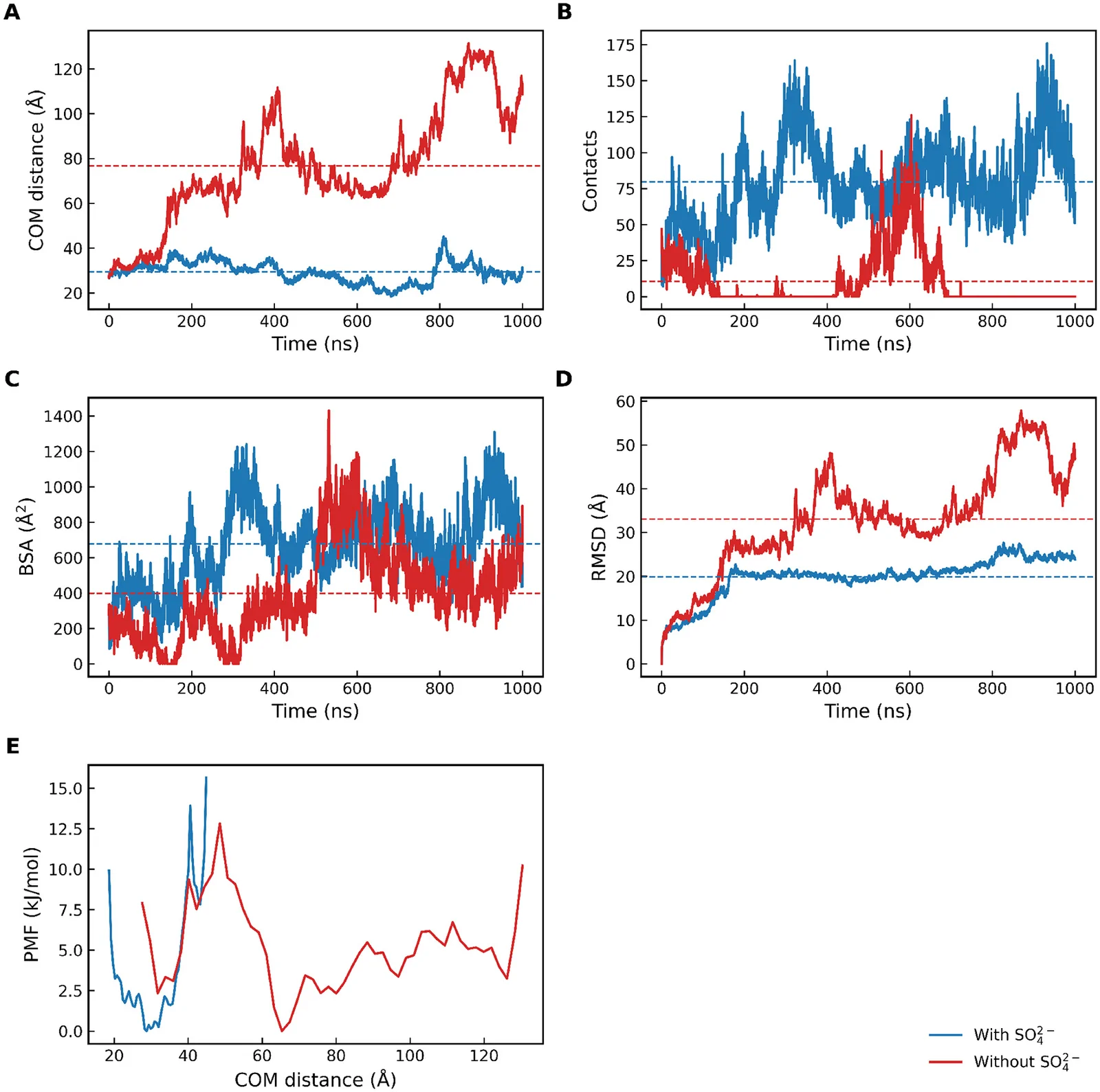
Comparison of structural and energetic properties of the CG Cnm–Cnm complex in the presence and absence of sulfate ions. **(A)** Time evolution of the center-of-mass (COM) distance between the two proteins. **(B)** Number of intermolecular contacts. **(C)** Buried surface area (BSA). **(D)** RMSD of the complex after alignment. **(E)** Potential of mean force (PMF) as a function of COM distance. Sulfate ions promote a stable bound state characterized by shorter distances, increased contacts, larger buried interface, reduced structural fluctuations, and a deeper free-energy minimum.

This behavior is consistent with the analysis of intermolecular contacts (Figure 5B). The sulfate-containing system exhibits a markedly higher number of contacts (79.52 on average) compared to the sulfate-free system (10.53), reflecting a tighter and more persistent interaction interface.

Further support is provided by the Buried Surface Area (BSA) (Figure 5C), which is an indicator of aggregation (higher BSA value, stronger aggregation tendency). In the presence of sulfate, the mean BSA reaches 676.45 Å^2^, whereas the sulfate-free system shows a significantly lower value (396.75 Å^2^). Importantly, the minimum BSA remains above 85 Å^2^ when sulfate is present, while it approaches zero in the control system, indicating transient dissociation events. Although maximum BSA values are comparable (1309.8 vs 1431.35 Å^2^), the sulfate-containing complex reaches high interface values earlier ( 200–300 ns) and maintains them with reduced fluctuations.

The RMSD analysis over the full 1 μs trajectory further highlights distinct dynamical regimes. In the presence of ${\text{SO}}_{4}^{2-}$, the RMSD stabilizes after an initial equilibration phase (~200 ns) around a mean value of 19.83 Å(Figure 5D), indicating a stable relative orientation between the two proteins. Conversely, in the absence of sulfate, the RMSD remains highly variable, oscillating between 30 Åand 60 Åwithout reaching a plateau, with peaks around 850 ns corresponding to nearly dissociated configurations.

These structural and dynamical observations are reflected in the free-energy landscape. The Potential of Mean Force (PMF) (see Equation 2) calculated from the COM distance (Figure 5E) shows a well-defined minimum in the presence of sulfate, whereas the sulfate-free system exhibits a flatter and more distributed profile.

To assess the dependence of these profiles on simulation time, the trajectories were further analyzed over four consecutive 250-ns intervals (Table 1). In the sulfate-containing system, the dominant PMF minimum remained within the low-distance region throughout the simulation, ranging from approximately 23.5 to 32.1 Å, with mean COM distances between 24.1 and 32.6 Å. In contrast, the sulfate-free system showed pronounced temporal variability and a progressive tendency toward larger intermolecular separations. After the first 250 ns, the dominant PMF minima shifted to approximately 66–126 Å, with the mean COM distance reaching 109.1 Å during the final 250 ns interval. These results indicate persistent close association in the sulfate-containing system, whereas in the absence of sulfate the proteins progressively explore more separated configurations.

**Table 1:** Time-window analysis of the COM-distance distributions used to assess the sampling dependence of the PMF profiles.

Time window (ns)	*r*_min_ (Å)	Mean COM (Å)	SD (Å)
With${\text{SO}}_{4}^{2-}$			
0–250	32.11	32.59	2.82
250–500	29.97	30.59	3.14
500–750	23.53	24.12	2.81
750–1000	28.89	30.11	4.93
Without${\text{SO}}_{4}^{2-}$			
0–250	31.43	47.48	15.14
250–500	80.38	79.55	12.59
500–750	66.40	70.62	6.59
750–1000	126.54	109.15	14.15

The position of the dominant PMF minimum (*r*_min_), mean COM distance, and corresponding standard deviation are reported for four consecutive 250-ns intervals.

The time-window analysis also indicates that the sampled distributions, particularly for the sulfate-free system, should not be regarded as fully converged equilibrium free-energy landscapes. Since the unbiased trajectories do not provide repeated equilibrium sampling of association and dissociation events, the PMF profiles are interpreted here as qualitative descriptors of the interaction regimes explored during the simulations rather than as absolute thermodynamic binding or dissociation free energies.

As shown in Figure 6, the AA trajectories reproduce the same trend observed in the CG simulations. In the presence of sulfate, the ligand remains consistently closer to the protein, as indicated by the lower minimum distance (Figure 6A), while maintaining a larger number of intermolecular contacts throughout the simulation (Figure 6B). Conversely, the sulfate-free system exhibits significantly larger fluctuations in the minimum distance and fewer and sparser intermolecular contacts, supporting a less stable binding mode. Representative snapshots from the all-atom simulations (Figure 7) qualitatively illustrate the different binding modes adopted by the two systems.

**Figure 6:**
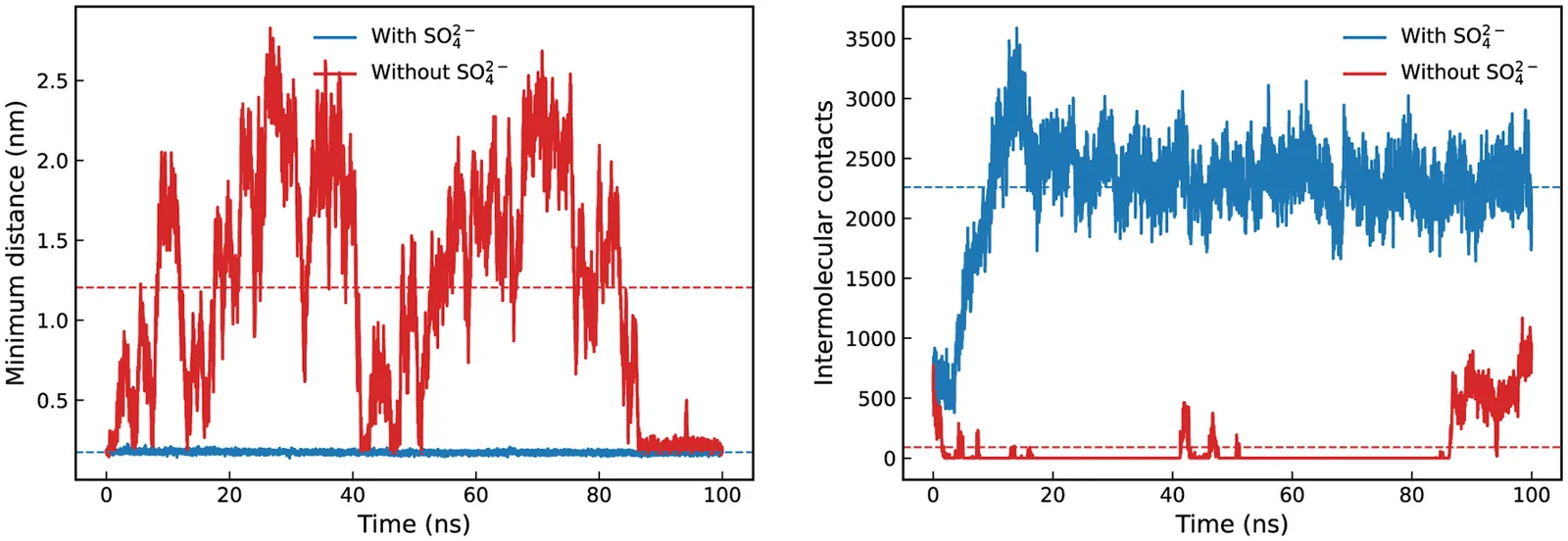
Comparison of structural and energetic properties of the AA Cnm–Cnm complex in the presence (blue) and absence (red) of sulfate ions. **(Left)** time evolution of the minimum distance between the ligand and the protein. **(Right)** time evolution of the number of intermolecular contacts. In both analyses, the sulfate-bound system shows a more stable interaction than the sulfate-free system.

**Figure 7:**
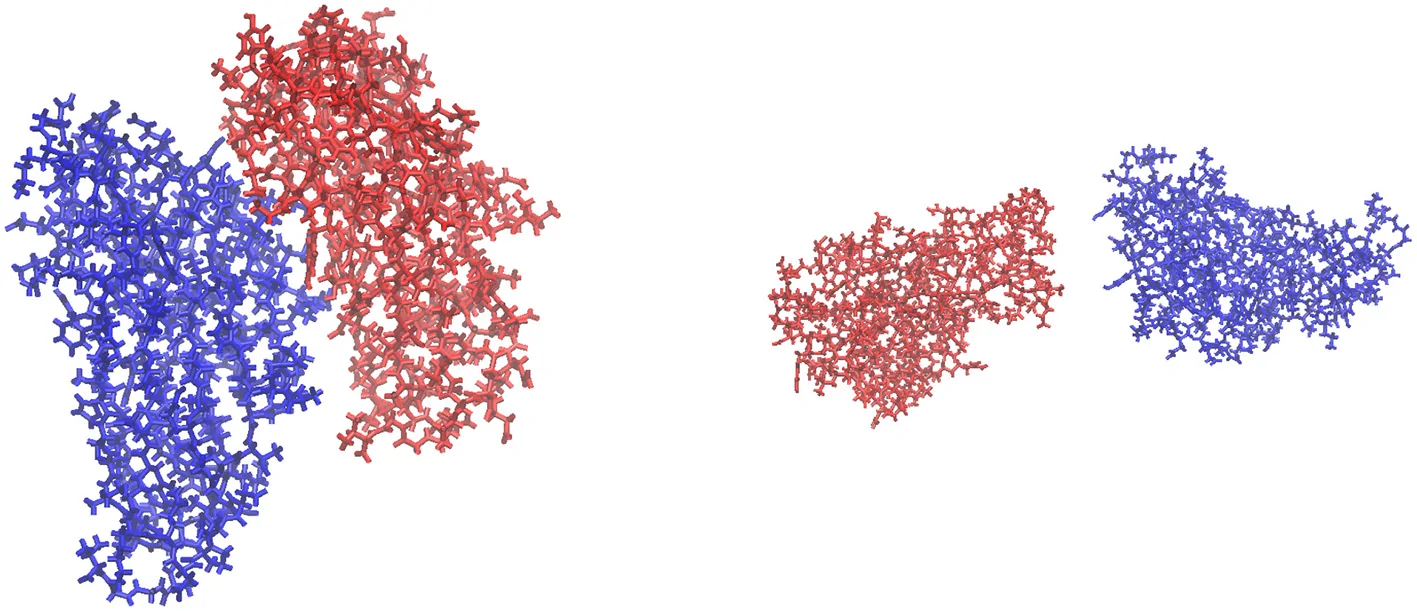
Representative snapshots of the Cnm–Cnm complex obtained from the all-atom molecular dynamics simulations in the presence **(left)** and absence **(right)** of sulfate ions. The two protein chains are shown in red and blue. The displayed conformations are representative of the bound state and qualitatively illustrate the different intermolecular arrangements observed during the simulations.

A residue-level contact analysis was performed to further characterize the Cnm–Cnm interaction interface. Residue pairs were considered to be in contact when their minimum heavy-atom distance was ≤ 4.5 Å. The analysis revealed an extended and highly persistent interaction interface, with 16 residue pairs maintaining contact in at least 70% of the analyzed trajectory frames (Table 2). Six pairs were particularly persistent, remaining in contact for more than 90% of the trajectory: Tyr91–Glu178 (98.13%), Thr78–Asn177 (96.63%), Gln73–Thr206 (94.57%), Asn69–Phe172 (91.66%), Gln65–Thr158 (90.54%), and Arg36–Glu178 (90.36%). These results, reported in Table 3, identify a stable network of residue–residue contacts underlying the Cnm–Cnm bound configuration and provide a residue-level characterization of the interaction interface.

**Table 2:** Persistent residue–residue contacts at the Cnm–Cnm interaction interface.

Monomer A	Monomer B	Contact frequency (%)	Mean distance (Å)
Tyr91	Glu178	98.13	3.56
Thr78	Asn177	96.63	3.02
Gln73	Thr206	94.57	3.34
Asn69	Phe172	91.66	3.47
Gln65	Thr158	90.54	2.94
Arg36	Glu178	90.36	2.89
Glu66	Arg170	86.72	2.96
Gln65	Gly159	82.69	3.68
Ile79	Tyr156	78.92	3.33
Thr78	Tyr156	78.33	3.62
Ile68	Tyr156	76.43	3.54
Ser80	Tyr156	75.11	3.74
Tyr91	Asn177	74.19	3.83
Gln73	Ser254	73.09	3.54
Arg36	Gly152	72.10	3.78
Asp50	Asp160	70.81	3.98

Residue pairs were considered in contact when their minimum heavy-atom distance was ≤ 4.5 Å.

Contact frequency represents the percentage of analyzed trajectory frames satisfying this criterion.

For clarity, only highly persistent residue pairs, defined here as those present in at least 70% of the trajectory frames, are reported.

The mean minimum distance was calculated over frames in which the corresponding contact was present.

**Table 3:** Comparison of protein–protein interaction metrics for simulations performed with and without sulfate ions.

Metric	With ${\text{SO}}_{4}^{2-}$	Without ${\text{SO}}_{4}^{2-}$
Mean COM distance (Å)	29.35 ± 4.74	76.70 ± 25.41
Mean number of contacts	79.52 ± 27.39	10.53 ± 18.62
Mean BSA (Å^2^)	676.45 ± 214.03	396.75 ± 248.40
Mean RMSD (Å)	19.83 ± 4.49	33.05 ± 11.68
Dissociation Δ*G* (kJ/mol)	10.34	5.73

Values are reported as mean ± standard deviation.

Taken together, our findings support the view that, under the simulated conditions, the presence of sulfate ions is associated with shorter intermolecular distances, increased contacts, larger buried surface area, and reduced structural fluctuations, all of which contribute to a deeper free-energy minimum. In contrast, the sulfate-free system samples more dispersed conformations, characteristic of a weakly bound or partially dissociated state. This scenario was to be expected. A similar pattern has been previously observed in both protein-protein aggregation (Spinozzi et al., 2002) and in the self-assembly of *fd*−viruses (Anop et al., 2025) in solutions with ions. However, because the present simulations compare only sulfate-containing and sulfate-free systems, they do not allow us to determine whether the observed stabilization is specific to ${\text{SO}}_{4}^{2-}$ or reflects a more general effect of divalent ions and the resulting modification of the local electrostatic environment. Simulations including other physiologically relevant ionic species under otherwise equivalent conditions will be required to establish the degree of sulfate specificity.

## 4 Conclusion

In this study, we integrated experimental observations from microscopy and Raman spectroscopy with multiscale molecular dynamics simulations to investigate molecular factors potentially contributing to Cnm-associated aggregation in *Streptococcus mutans*. The experimental results reveal biochemical and structural differences between Cnm-positive and Cnm-negative strains, including a greater aggregation propensity and differences in Raman spectral features associated with sulfur-containing species in the Cnm-positive phenotype. Because whole-cell Raman spectroscopy reflects the overall biochemical composition of the bacterial cell, these spectral differences cannot be attributed specifically to the Cnm protein and may also reflect broader phenotypic differences between the investigated strains. At the molecular level, our simulations provide a possible mechanistic interpretation to be considered alongside these experimental observations. Under the investigated simulation conditions, both all-atom and coarse-grained simulations consistently show greater stability of the Cnm–Cnm complex in the presence of sulfate. This stabilization is reflected in reduced inter-protein distances, increased intermolecular contacts, larger buried surface areas, and a deeper free-energy minimum. The consistency between the atomistic and coarse-grained descriptions supports the observation of sulfate-associated stabilization across the two simulation resolutions, while the calculated free-energy profiles should be interpreted as qualitative descriptors of the sampled interaction landscape rather than as absolute thermodynamic free energies. Taken together, the experimental and computational results support a scenario in which the ionic environment may influence Cnm–Cnm association. It is however important to remark that the present study does not provide direct experimental evidence that sulfate promotes aggregation of Cnm-positive *S. mutans* cells, nor does it establish that the sulfur-containing species detected by Raman spectroscopy originate from Cnm. The sulfate-dependent stabilization observed in the simulations should therefore be regarded as a molecular hypothesis that may contribute to explaining the enhanced aggregation phenotype, and that requires direct experimental validation under controlled ionic conditions. Furthermore, because the simulations compare only sulfate-containing and sulfate-free systems, they do not establish whether the observed stabilization is specific to sulfate or reflects a more general effect of divalent ions or changes in the local electrostatic environment.

Several computational limitations of the present study should also be acknowledged. The computational model is restricted to the protein level and describes the association of two isolated Cnm molecules in a simplified aqueous environment. It therefore does not reproduce the spatial organization and molecular complexity of the *S. mutans* cell surface, where Cnm is embedded within a heterogeneous bacterial envelope and may interact with other surface components, nor does it account for multispecies microbial communities, host proteins, or the complex physicochemical conditions of the oral microenvironment. Furthermore, the simulations do not directly model bacterial adhesion to vascular tissues or the pathogenesis of cerebrovascular disease. Consequently, the enhanced Cnm–Cnm stability observed in the presence of sulfate should be interpreted as a molecular mechanism that may contribute to the aggregation phenotype of Cnm-positive strains under the investigated conditions, rather than as a direct representation of bacterial aggregation *in vivo* or evidence of a causal mechanism linking Cnm-positive *S. mutans* to stroke. Within these limitations, Cnm–Cnm association may represent one molecular component of the broader biological processes associated with the systemic effects of Cnm-positive strains.

Future work will aim to extend these findings by investigating the role of other physiologically relevant ions, assessing the effect of sequence and structural variation among Cnm variants, identifying the specific molecular components responsible for the observed Raman spectral differences and distinguishing direct Cnm contributions from broader Cnm-associated phenotypic changes, exploring larger multimeric assemblies, and integrating host factors such as collagen and endothelial components to better capture the complexity of *in vivo* conditions.

## Data Availability

The original contributions presented in the study are included in the article/supplementary material, further inquiries can be directed to the corresponding authors.
